# Novel carbon nanozymes with enhanced phosphatase-like catalytic activity for antimicrobial applications

**DOI:** 10.1186/s11671-023-03856-y

**Published:** 2023-05-23

**Authors:** Lazzat Nurtay, Enrico Benassi, Faisal Nazir, Dana Dastan, Assem Utupova, Adilet Dautov, Kanat Dukenbayev, Yingqiu Xie, Tri T. Pham, Haiyan Fan

**Affiliations:** 1 Department of Chemistry, School of Sciences and Humanities, Nazarbayev University, Qabanbay Batyr 53, Nursultan, 010000 Kazakhstan; 2 Novosibirsk State University, Pirogova Str. 2, Novosibirsk, Russia 630090; 3 Department of Biology, School of Sciences and Humanities, Nazarbayev University Nazarbayev University, Qabanbay Batyr 53, Nursultan, 010000 Kazakhstan; 4 Department of Electrical and Computer Engineering, School of Engineering and Digital Sciences, Nazarbayev University Nazarbayev University, Qabanbay Batyr 53, Nursultan, 010000 Kazakhstan

**Keywords:** Carbon-based nanoparticles, Enzymatic activity, Phosphatase and antimicrobial properties, Minimum inhibitory concentration

## Abstract

**Abstract:**

In this work, Sulfur and Nitrogen co-doped carbon nanoparticles (SN-CNPs) were synthesized by hydrothermal method using dried beet powder as the carbon source. TEM and AFM images indicated that these SN-CNPs form a round-shape ball with an approximate diameter of 50 nm. The presence of Sulfur and Nitrogen in these carbon-based nanoparticles was confirmed by FTIR and XPS analyses. These SN-CNPs were found to have strong phosphatase-like enzymatic activity. The enzymatic behavior of SN-CNPs follows the Michaelis–Menten mechanism with greater *v*_*max*_ and much lower *K*_*m*_ values compared to *alkaline phosphatase*. Their antimicrobial properties were tested on *E. coli* and *L. lactis*, with MIC values of 63 μg mL^−1^ and 250 μg mL^−1^, respectively. SEM and AFM images of fixed and live *E. coli* cells revealed that SN-CNPs strongly interacted with the outer membranes of bacterial cells, significantly increasing the cell surface roughness. The chemical interaction between SN-CNPs and phospholipid modeled using quantum mechanical calculations further support our hypothesis that the phosphatase and antimicrobial properties of SN-CNPs are due to the thiol group on the SN-CNPs, which is a mimic of the cysteine-based protein phosphatase. The present work is the first to report carbon-based nanoparticles with strong phosphatase activity and propose a phosphatase natured antimicrobial mechanism. This novel class of carbon nanozymes has the potential to be used for effective catalytic and antibacterial applications.

**Graphical abstract:**

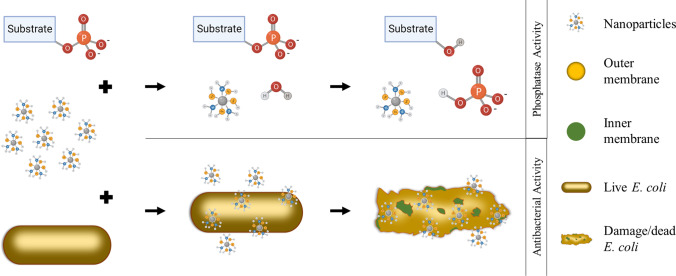

**Supplementary Information:**

The online version contains supplementary material available at 10.1186/s11671-023-03856-y.

## Background

The concept of “artificial enzyme” was first introduced by Breslow [1], whose work showed that the enzymatic function could be achieved by attaching a metal catalytic group to a hydrophobic macromolecular cavity. The efforts of synthesizing such an artificial enzyme have, ever since, been focused on reducing the mobility of the active sites and constraining the molecular conformation to ensure the cooperativity, which is essential for the enzymatic functioning in biological systems [2–4]. Borrowing such an idea, radical scavenging features were identified in polyhydroxylated and carboxylate fullerenes as the mimic of superoxide dismutase (SOD) [5, 6]. The concept of artificial enzyme was further extended to nanomaterials, and the naming of “nanozyme” was then proposed [7–11]. For nanozymes, the key components to establish a cluster of functional groups are either through surface modification or self-assembly, allowing multivalent interactions with the receptors [12–14].

Ever since the discovery of nanozyme, a variety of nanomaterials has been synthesized to fulfill various enzymatic functions, such as peroxidase (POD) [15–17], oxidase (OD) [18], SOD [19, 20], nuclease [21, 22] and phosphatase [23, 24]. Overall, nanozymes may be classified into two main groups, namely: the metal and the carbon-based ones. However, the carbon-based nanozymes reported so far have only exclusively shown POD activity [25–28].

In our previous work, Nitrogen-doped carbon dots (CDs) derived from beet were found to participate in the protein tyrosine dephosphorylation through binding to the phosphate group [29]. Recently, N-doped CDs (N-CNPs) were reported as promising materials for analytical methods, being able to quantitatively determine *alkaline phosphatase* (ALP) based on a fluorescence turn-of detection approach [30]. The present work aims to develop the Sulfur and Nitrogen co-doped nanoparticles (SN-CNPs) that possess phosphatase activity mimicking cysteine-based protein phosphatase. To the best of our knowledge, the only reported nanozyme showing phosphatase activity was inorganic-based, i.e. Cerium (IV) oxide nanoparticles [23]. We attempted to develop a carbon-based nanozyme with phosphatase activity in this work by utilizing benefits like low cost, low toxicity, greater flexibility, high stability, and excellent electronic/optical properties [31]. Exploiting the mechanism of protein phosphatase, SN-CNPs were synthesized using beet as a raw material in combination with Sulfur and Nitrogen sources. The ability of dephosphorylation of these SN-CNPs was detected through nitro-blue tetrazolium and 5-bromo-4-chloro-3’-indolyphosphate (NBT-BCIP) and para-nitrophenylphosphate (pNPP) substrates.

On the other hand, antimicrobial properties of carbon-based nanoparticles (CNPs) including CDs without Sulfur doping have been widely explored [32–38]. Three main antimicrobial mechanisms using such materials were proposed: (1) due to the peroxidase activities of CNPs and, consequently, the production of reactive oxidation species (ROS) [32, 37, 38]; (2) caused by the charge, especially positive, carried on the surface of CNP [33, 36]; (3) induced by polymer passivation reagents either increasing mechanical stress to cell membrane or carrying special functional groups that potentially destroy bacterial biofilm [34, 35]. Despite the progress made in the enhancement of antimicrobial effects and drug resistance reduction, the mechanistic details of antimicrobial effects induced by CNPs remain elusive. Moreover, developing CNPs-based antimicrobial reagents possessing high sensitivity and selectivity against different types of bacteria is highly desired.

In the history of antibiotics, the Enterobacteriaceae family developed the strongest drug resistance. *E. coli* is a typical representative in this bacteria family with unique transmission pattern and was the most studied bacterium most probably because it can attack various anatomical sites in the human body [39]. Due to its double membranes structure, namely the outer membrane (OM) and inner membrane (IM), and cell wall in between, *E. coli* showed more resistant than other Gram-positive bacteria towards antibiotics that were designed to attack the cell wall, not to mention its multi-drug resistance.

Some antibiotics were designed to target the OM of the Gram-negative bacteria. For instance, polymyxin B, carrying positive charge, was reported to protrude the OM by interacting with a negatively charged phosphate group on Lipid A located on the OM [40]. Other kinds of antibiotics attack OM by inhibiting the synthesis of membrane RNA and proteins [41]. In this work, we attempted to develop carbon-based nanoparticles with enzymatic activities using beet, a low cost, environmental friendly and safe nature product, as the carbon source. In particular, we anticipated that the addition of sulfur would result in the production of a nanozyme with phosphatase activity that mimicked cysteine-based protein phosphatase. Given the molecular makeup of the cell wall and membrane of bacteria, we intended to introduce a new antimicrobial approach in which the bactericidal effect will be achieved by hydrolyzing phospholipids on the OM and IM as well as the phosphate cross link in the cell wall. The present work proposes for the first time a phosphatase-based antimicrobial mechanism by carbon-based nanozyme.

In order to understand the mechanism of interaction energies between SN-CNPs and phospholipid on the OM, quantum mechanical calculations were performed using methanethiol and dimethyl phosphate to model the active functional group –SH on SN-CNPs and the phospholipids, respectively. The computed interaction energies and changes in vibrational spectra of possible conformations will further corroborate our hypothesized mechanism.

## Results and discussion

### Chemical identity and morphology of SN-CNPs

**Fig. 1:**
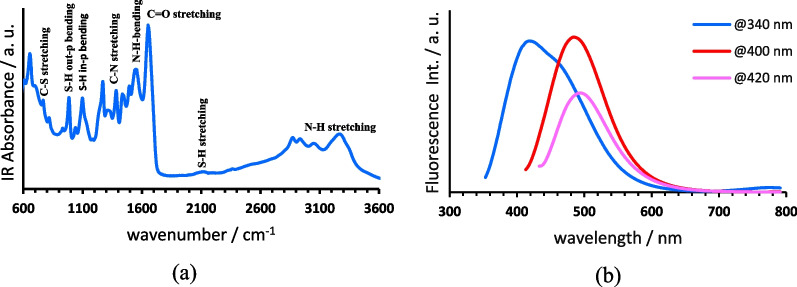
(**a**) FT-IR spectra of dried SN-CNPs, (**b**) Fluorescence spectra of the aqueous solution of SN-CNPs upon excitation at 340 nm (blue), 400 nm (red) and 420 nm (pink), respectively

FT-IR spectrum of SN-CNPs shown on Fig. 1a display the amide group absorption pattern with the absorption bands centered at 1630 cm^−1^ representing the C=O stretching, 1560 cm^−1^ is the N–H in-plane bending, 1280 cm^−1^ is C–N stretching and 3250 cm^−1^ is the N–H stretching. Meanwhile, C–S and S–H functional groups are characterized by the absorption bands at 764 cm^−1^ signifying C–S stretching, those centered at 987 cm^−1^ and 1098 cm^−1^ corresponding to S–H in-plane and out-of-plane bending, respectively, and 2385 cm^−1^ for S–H stretching vibration [42]. From a synthetic point of view, formation of S–H and C–S bonds through hydrothermal reaction is possible especially when both ethylenediamine and elemental Sulphur are among the precursors [43]. On the other hand, disulfide bond is an essential byproduct for the reaction between ethylenediamine and elemental sulfur, which enables the production of deprotonated thiol through thiol-disulfide exchange reaction [44].

The photoluminescence of SN-CNPs was measured and compared using fluorescence spectroscopy. Upon excitation at 340 nm, the fluorescence spectrum of SN-CNPs can be deconvolved into three peaks at 424 nm, 458 nm and 495 nm, respectively, corresponding to three distinguishable fluorophores (Fig. 1b). The attached photo depicts a blue emitted vial containing aqueous solution of SN-CNPs upon photoexcitation by a UV-lamp at 365 nm wavelength. At the excitation wavelength of 400 nm and 420 nm, fluorescence is only observed at 458 nm and 495 nm, respectively. Overall, the photoluminescence does not show any excitation wavelength dependence. This means that the content of C–O–H and C–O–C groups in SN-CNPs is relatively low and it agrees well with the observations from FT-IR spectrum. According to Liu et al., [30] these functional groups lead to the generation of many localized states within n-π* and π-π* gap, which produces multiple paths for the radiative decay.

The measured zeta potential of SN-CNPs diluted in water at low concentration (50 μg mL^–1^) indicates a potential peak at –8.32 mV (see Fig. S1). This result suggests that SN-CNPs are slightly negatively charged, which is opposite to the previously observed positively charged beet-derived N-CNPs without S-doping [29]. This observation further confirms the presence of the thiol functional group –SH because the addition of Sulfur fundamentally changes the charge distribution on the surface of N-CNPs from positive to negative.

**Fig. 2:**
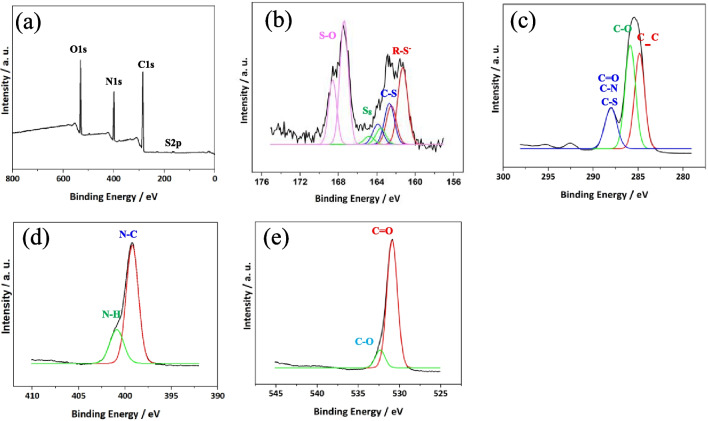
High resolution XPS spectra of dried SN-CNPs. (**a**) Survey spectrum for SN-CNPs; (**b**) S(2*p*); (**c**) C(1* s*); (**d**) N(1*s*); and (**e**) O(1*s*)

To further characterize the hybridisation state of the surface functional groups, XPS spectra were collected for SN-CNPs with the atomic ratio of N(22.2%), C(58.8%), O(18.4%) and S(0.66%) (Fig. 2). The survey spectra showed the characteristic peaks of S(2*p*) present for SN-CNPs, indicating a successful doping of Sulfur. In the high resolution spectrum, six peaks including three ^2^P_3/2_ peaks at 161.3 eV, 162.7 eV, and 163.6 eV along with their doublet partners, three ^2^P_1/2_ peaks at 162.5 eV, 163.9 eV and 164.8 eV respectively with half intensity relative to corresponding ^2^P_3/2_ peaks. The peak with the binding energy (BE) of 161.3 eV (^2^P_3/2_) was generally characterized as deprotonated thiol group R–S^−^, the peak at 162.7 eV (^2^P_3/2_) stands for the S in S–C contact, whereas peak at 163.6 eV (^2^P_3/2_) refers to the neutral sulfur, which is consistent with the FT-IR result. On the other hand, the sub-band at higher energy with two peaks corresponding to ^2^P_3/2_ at 167.4 eV and ^2^P_1/2_ at 168.6 eV was assigned as Sulfur existing as oxy-sulfur (S–O), which might be caused by the oxidation of Sulfur during hydrothermal reaction [45–48].

The deconvolved peak at 284.8 eV was assigned to the C(1*s*) state in the form C–C, the one at 285.9 eV to C–O, likely originated from the poly-sugar component in beet. The sub-band at higher energy could be deconvolved into peaks at 287.9 eV representing C=O, C–N, C–S and 288.7 eV representing O–C=O [49] Meanwhile, the high resolution XPS spectra for C (1*s*) shows two minor peaks with BE of around 293 eV and 295 eV. In general, it is assumed that the C in the form of CF_x_- or carbonate in polymer is represented by the C 1*s* peak with BE at 293 eV. However, the synthesis of CNPs did not utilize F, F-related reagents, or carbonate. Furthermore, the presence of both peaks at 293 eV and 295 eV, as well as their relative intensities, suggests that these two peaks are caused by surface contamination with K. In addition, the quantitative information of peak area indicated the atomic percentage of Sulfur in the solid product of SN-CNPs was ~ 0.66% [50, 51].

**Fig. 3:**
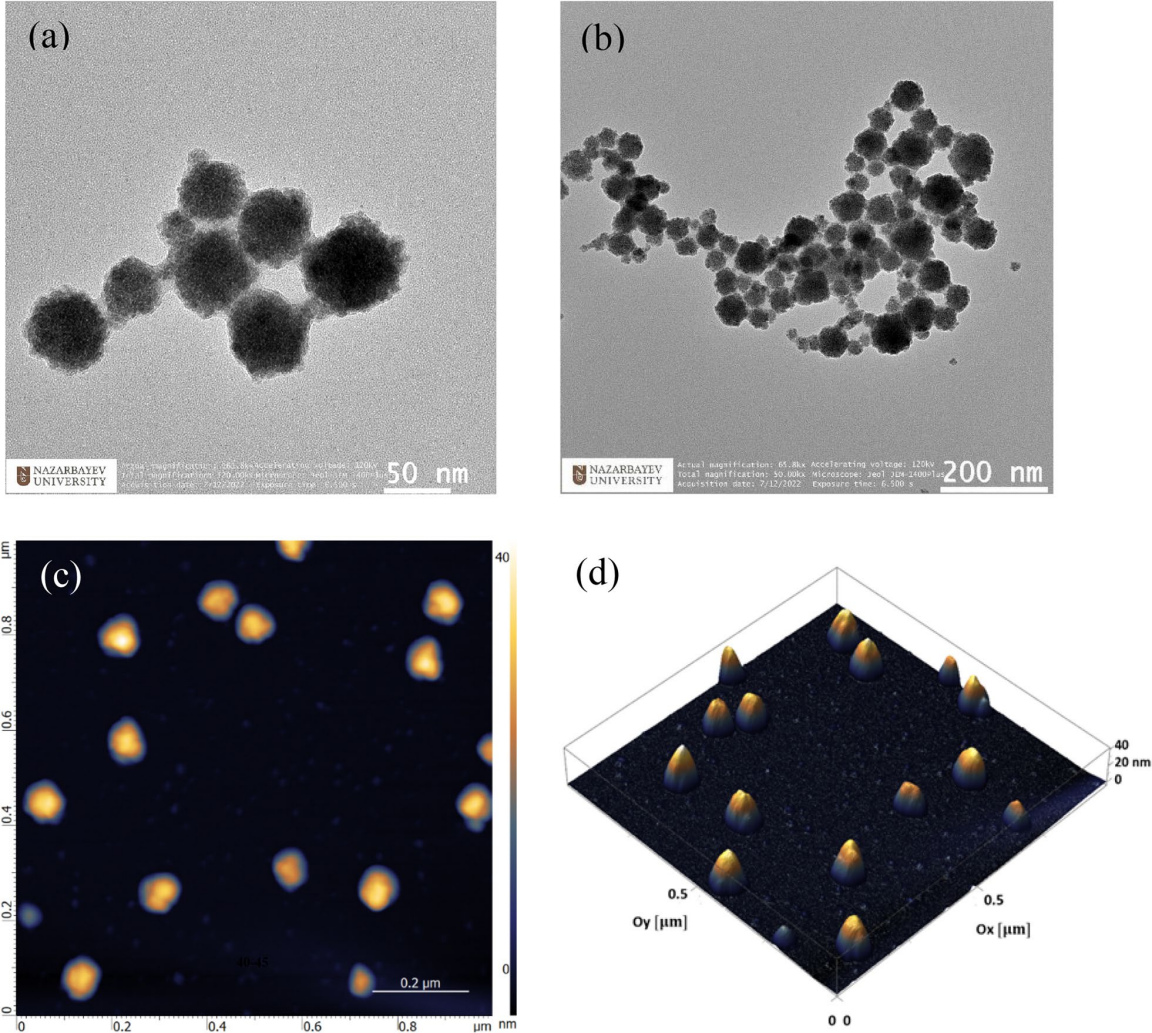
TEM (**a**, **b**) and AFM (**c**, **d**) images of dried SN-CNPs prepared from 100 μg mL^−1^

The morphology and the size of SN-CNPs were characterized using transmission electron microscopy (TEM) and atomic force microscopy (AFM) imaging. The TEM images revealed a structure of a round-shaped particles with an average diameter of 50 nm at 100 μg mL^−1^ concentration (Fig. 3a, b and Figure S2). The TEM images are comparable to images of CNPs with the similar sizes reported in the literature [52]. According to the AFM 2D image (Fig. 3c), the majority of particles have a diameter of around 40–50 nm, whereas the AFM 3D image (Fig. 3d) indicates that their heights varied between 30 and 40 nm. Such morphology often appeared in a concentration range up to 100 μg mL^−1^ and their shape and dimension seems to depend on the concentration for concentration above 100 μg mL^−1^, wherein the average diameter as well as the height of the particles increased with increasing concentration due to coalescence (see Fig. S3 for more details).

### Phosphatase activity assay

**Fig. 4:**
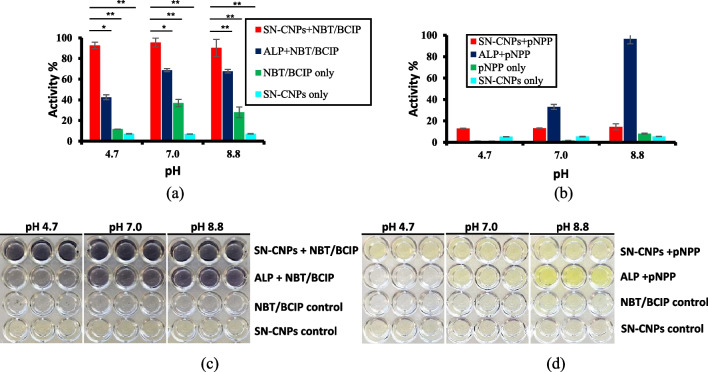
Phosphatase assay for SN-CNPs in comparison with that of ALP at 25 °C. **a**, **c** NBT–BCIP substrate; **b**, **d** pNPP substrate. Error bars indicate standard deviation upon three trials

The aqueous solution of SN-CNPs exhibited phosphatase activity with NBT-BCIP as a substrate at pH values of 4.7, 7.0 and 8.8 as shown in Fig. 4a and c, which was nearly independent of pH. At all three aforementioned pH values, representing weak acidic, neutral and weak basic conditions, respectively, the phosphatase activity of SN-CNPs was much stronger than that of ALP, whose strongest activity was shown at pH 8.8.

Regardless of the strong phosphatase activity of SN-CNPs indicated by NBT-BCIP substrate, much weaker sensitivity was observed with pNPP substrate compared to ALP (Fig. 4b and d). Such a result was hypothesized due to the strong interaction between sulfhydryl or thiol group and nitro group [53, 54]. While weak sensitivity of the phosphatase was generally offered by SN-CNPs with pNPP substrate, stronger phosphatase activity than ALP was identified at pH 4.7.

In our previous study [29], the interaction between positively charged amide groups and phosphate groups led to some weak phosphatase activity. However, the strong phosphatase activity exhibited by SN-CNPs might likely follow a similar mechanism of protein phosphatase. This was supported by both FT-IR and XPS characterizations, which revealed the existence of C–S–H functional groups on the surface of SN-CNPs. Despite the fact that SN-CNPs contain only ~ 0.66% Sulfur, they act as catalysts rather than reactants because the thiol group can be recovered through hydration after dephosphorylation. On the other hand, free cysteine and non-Sulfur or Nitrogen only doped CNPs (N-CNPs) failed to demonstrate significant phosphatase activity (Figure S4 & S8).

### Kinetic characterization

**Fig. 5:**
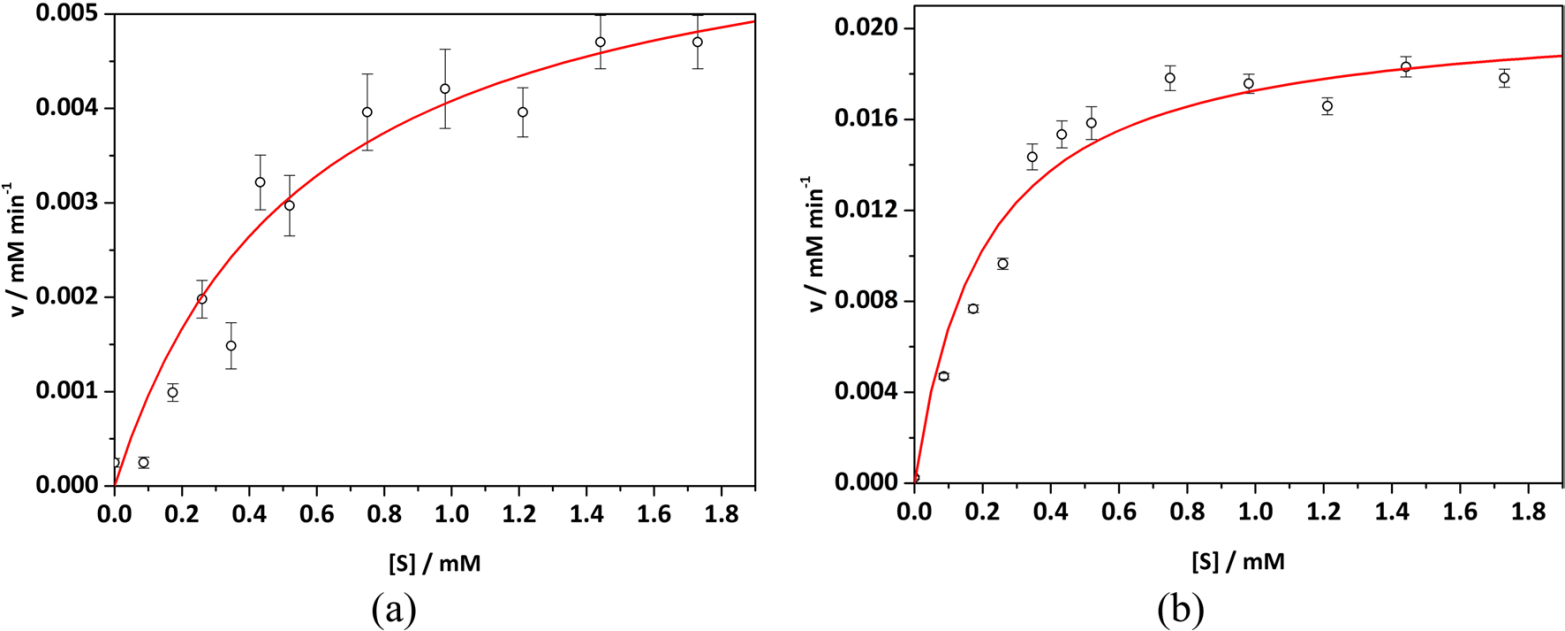
Rates of dephosphorylation (v, circles) at different concentrations of substrate (NBT-BCIP) along with the fitting results (red solid curve). (**a**) Calf-intestinal ALP and (**b**) SN-CNPs. Error bars indicate standard deviations upon three trials

The kinetic tests indicated that the phosphatase of SN-CNPs followed the Michaelis–Menten mechanism, which is similar to ALP (Fig. 5). Using NBT-BCIP as the substrate, the reaction rates were measured at different concentrations of BCIP at pH = 7 and T = 25 °C. The values of *v*_*max*_ and *K*_*m*_ were extracted from the non-linear least-square regression fit of the data and are shown in Table 1. Here, *v*_*max*_ represents the maximum rate achieved by the system and the Michaelis constant *K*_*m*_ is the substrate concentration at which the reaction rate is half of the *v*_*max*_. The Michaelis–Menten catalytic mechanism implies that SN-CNPs acted as catalysts in the dephosphorylate reaction. The catalytic properties of SN-CNPs can be explained in three different aspects: (1) the phosphatase activity was shown with NBT-BCIP substrate at a very low concentration of SN-CNPs (~ 20 μg mL^−1^ at pH 7) without further dependence of SN-CNPs concentration; (2) the dimension and morphology of the SN-CNPs, as well as the even distribution of Sulfur made each particle a reaction platform to ensure a multivalent interaction with the receptors; (3) SN-CNPs had a much lower *K*_*m*_ value (0.21 ± 0.01 mM) than ALP (0.56 ± 0.15 mM), demonstrating that SN-CNPs have a substantially stronger affinity for the BCIP substrate. Finally, at 25 °C, SN-CNPs had a considerably higher *v*_*max*_ value (20.82 ± 1.36 μM min^−1^) than ALP (6.38 ± 0.51 μM min^−1^), which is consistent with the higher phosphatase activity of SN-CNPs previously mentioned.

**Table 1:** The Michaelis–Menten mechanism’s parameters for SN-CNPs and Calf-intestinal ALP at T = 25 °C and pH = 7, using NBT-BCIP substrate, upon non-linear least-square regression

	*K*_*m*_/mM	*v*_*max*_/(μM min^−1^)	R^2^
SN-CNPs	0.21 ± 0.01	20.82 ± 1.36	0.99
ALP	0.56 ± 0.15	6.38 ± 0.51	0.99

### Antimicrobial effects of SN-CNPs against *E. coli* and *L. lactis* and working mechanism

**Fig. 6:**
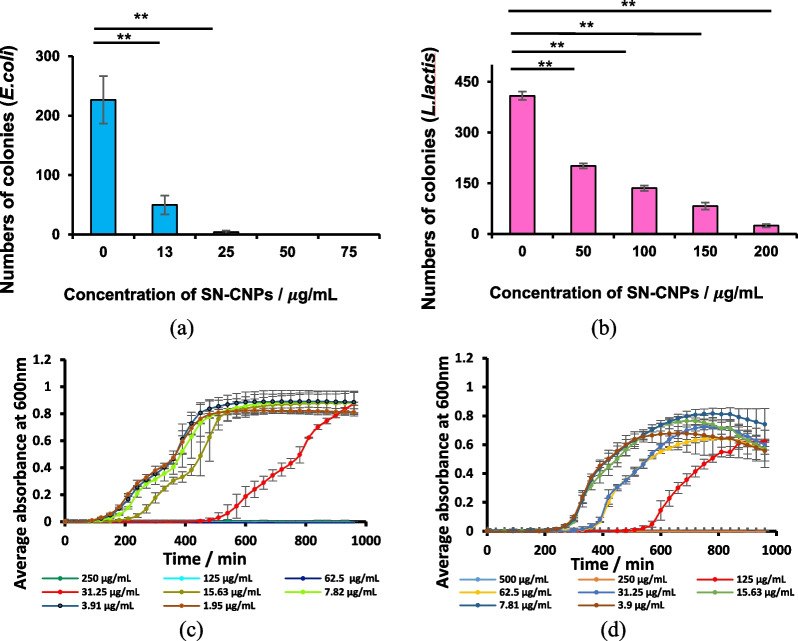
Antimicrobial tests: **a** SN-CNPs against *E. coli*; **b** SN-CNPs against *L. lactis*; **c** micro-dilution test of SN-CNPs against *E. coli*, and **d** micro-dilution test of SN-CNPs against *L. lactis*. Error bars indicate standard deviations from three trials

The antimicrobial effects of SN-CNPs were tested against Gram-negative bacteria *E. coli* (Fig. 6a) and Gram-positive bacteria *L. lactis* (Fig. 6b). The figure clearly shows that only 13 μg mL^−1^ of SN-CNPs is required to inhibit more than 50% of the control colonies of *E. coli* while at least 50 μg mL^−1^ is needed for *L. lactis.* Thus, SN-CNPs exhibited stronger inhibition against *E. coli* than *L. lactis*. Meanwhile, in order to achieve the similar bactericidal rate, the system requires a much higher concentration of non-Sulfur doped CNPs, as shown in Figure S5. This observation highlights the significance of the thiol group and the phosphatase activity it induces. A broth micro-dilution assay using OD600 plate reader was adopted to identify the minimum inhibitory concentration (MIC) for SN-CNPs against *E. coli* (Fig. 6c) and *L. lactis* (Fig. 6d). The MIC values of 63 μg mL^−1^ and 250 μg mL^−1^ were obtained for *E. coli* and *L. lactis*, respectively [55, 56]. Visual inspection of the bacteria growth on the agar plates further confirmed our results for the MIC because very few or almost no colonies were detected for concentrations above the determined MIC values as shown in Figure S6.

Previous study has shown that jute-derived, Sulfur containing carbon dots (CDs) also have antibacterial effects against *E. coli* and *S. aureus*. The authors attributed the antimicrobial effect to the strong interaction between cationic featured CDs and cell membrane/wall components [57]. The present work, however, characterized a negative potential for the aqueous solution of SN-CNPs, the bactericidal mechanism based on positive charge is therefore excluded.

**Fig. 7:**
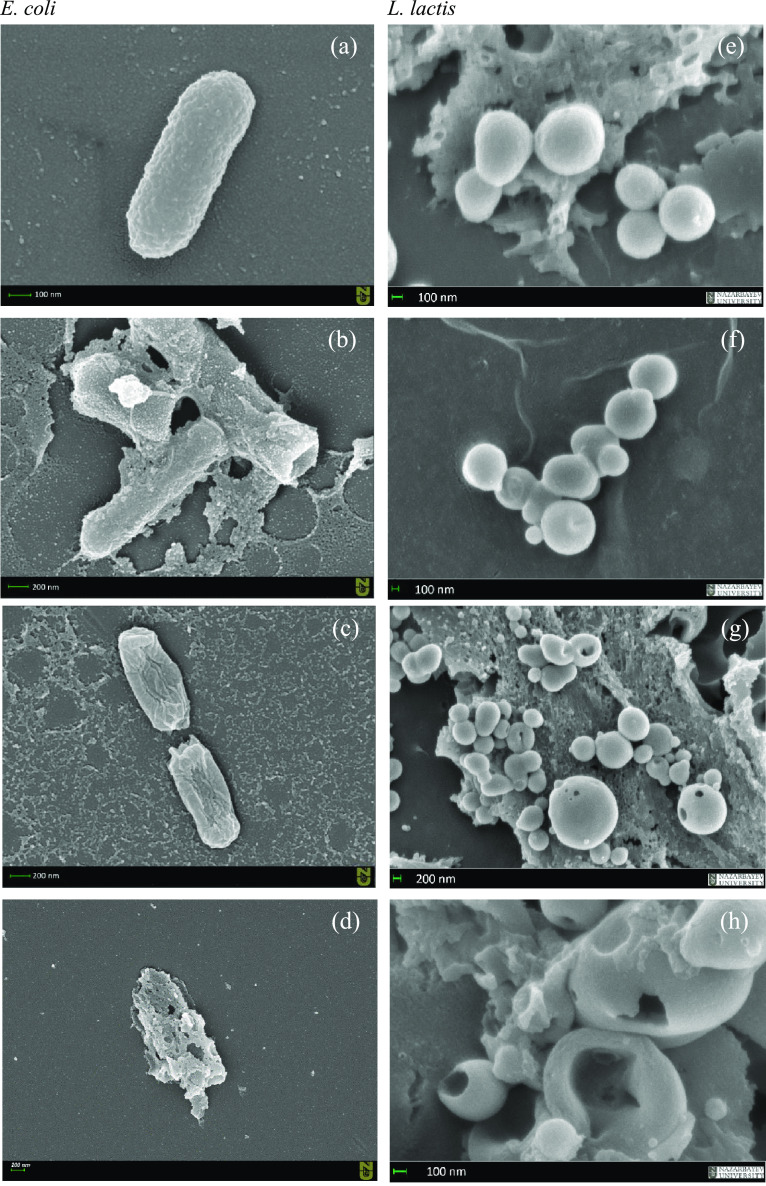
SEM images of *E. coli* (left panels; concentration of SN-CNPs: 50 μg mL^−1^) and *L. lactis* (right panels; concentration of SN-CNPs: 100 μg mL^−1^) upon treatment with SN-CNPs. **a**, **e** Control; **b**, **f** after 30 s; **c**, **g** after 30 min; **d**, **h** after 2 h

Lamb et al. emphasized that advanced microscopy techniques such as SEM and TEM imaging can shed light on the antimicrobial mechanisms by revealing the alteration in morphology and ultrastructure of bacterial cells upon treatment with antimicrobial reagents [58]. Following such a strategy, SEM images of both *E. coli* and *L. lactis* were acquired upon treatment of SN-CNPs at a concentration of 100 μg mL^−1^ for three different incubation periods (Fig. 7). SEM images of *E. coli* (left panel) and *L. lactis* (right panel) were taken at 30 s, 30 min and 2 h after treatment with SN-CNPs. The OM of *E. coli* cells were slightly peeled off when immersed in SN-CNPs solution for 30 s (Fig. 7b). For 30 min incubation, blebs along with the partial decay of the cell wall were observed (Fig. 7c). The appearance of blebs was interpreted as a sign of disruption occurring on the OM of Gram-negative bacterial cells either induced by positively charged polymyxin B molecules or membrane metabolism related RNA or protein synthesis inhibitors [59]. However, the damage on *E. coli* caused by SN-CNPs did not stop at the OM. After a 2 h treatment, the cell wall as well as the IM were destroyed (Fig. 7d), and the cells lost their structural integrity.

For *L. lactis* cells, within 30 s of treatment by SN-CNPs, negligible damages were observed on the cell wall (Fig. 7f). After 30 min, holes of different sizes started to appear, while the overall structure of the cell remained intact (Fig. 7g). After a 2 h-incubation, part of the cell wall fell apart and the cell became a hollow shell, indicating the leakage of the cytoplasm (Fig. 7h). The SN-CNPs are largely invisible in the cell SEM images in Fig. 7 due to the addition of the fixation reagent.

The morphological alteration at different stages observed on *E. coli* upon treatment with SN-CNPs indicated that cell death was initiated by the disruption of the OM. Such disruption observed in SEM images appears in two forms; peeled OM observed upon a 30 s treatment and blebs appeared upon a 30 min treatment. The peeled OM is likely due to the phosphatase of SN-CNPs, which leads to the dephosphorylation of the phospholipid located on the out layer of the OM and further disintegrated part of or the entire OM. While studying the role of phospholipids in the function of *E. coli*, Vitrac et al. realized that the alteration in phospholipid composition might bring a dramatic impact on the structure and function of the OM and even the entire cell [60]. The appearance of blebs, on the other hand, may be attributed to the negative potential of SN-CNPs, which potentially develops repulsion to the negatively charged OM and causes the uneven distribution of OM over the peptidoglycan.

Other than impairing the OM of *E. coli*, SN-CNPs were also observed to damage the cell wall of both *E. coli* and *L. lactis* indicated as holes on the cell structure shown in the SEM images. Lipoteichoic acid (LPS), a major component of the cell walls of Gram-positive bacteria, was found subjected to dephosphorylation by alkaline phosphatase [61]. On the other hand, phosphate esters were considered the backbone of *E. coli* cell walls [62]. Hence, the destruction exerted by SN-CNPs on the cell walls of both *E. coli* and *L. lactis* might be due to their phosphatase activities. It is important to note that, contrary to many other nanoparticles, SN-CNPs do not directly induce intracellular ROS. This result therefore provides further proof that the attack on bacterial membranes and cell walls by SN-CNPs via phosphatase activity results in antibacterial activity rather than the production of intracellular ROS (see Fig. S7). To emphasize the role of phosphatase instead of thiol group in the antimicrobial effect, an independent test using free cysteine containing the same amount of thiol relative to SN-CNPs was carried out, such a system failed to show phosphatase activity and antimicrobial effect against *E. coli* (Fig. S8).

**Fig. 8:**
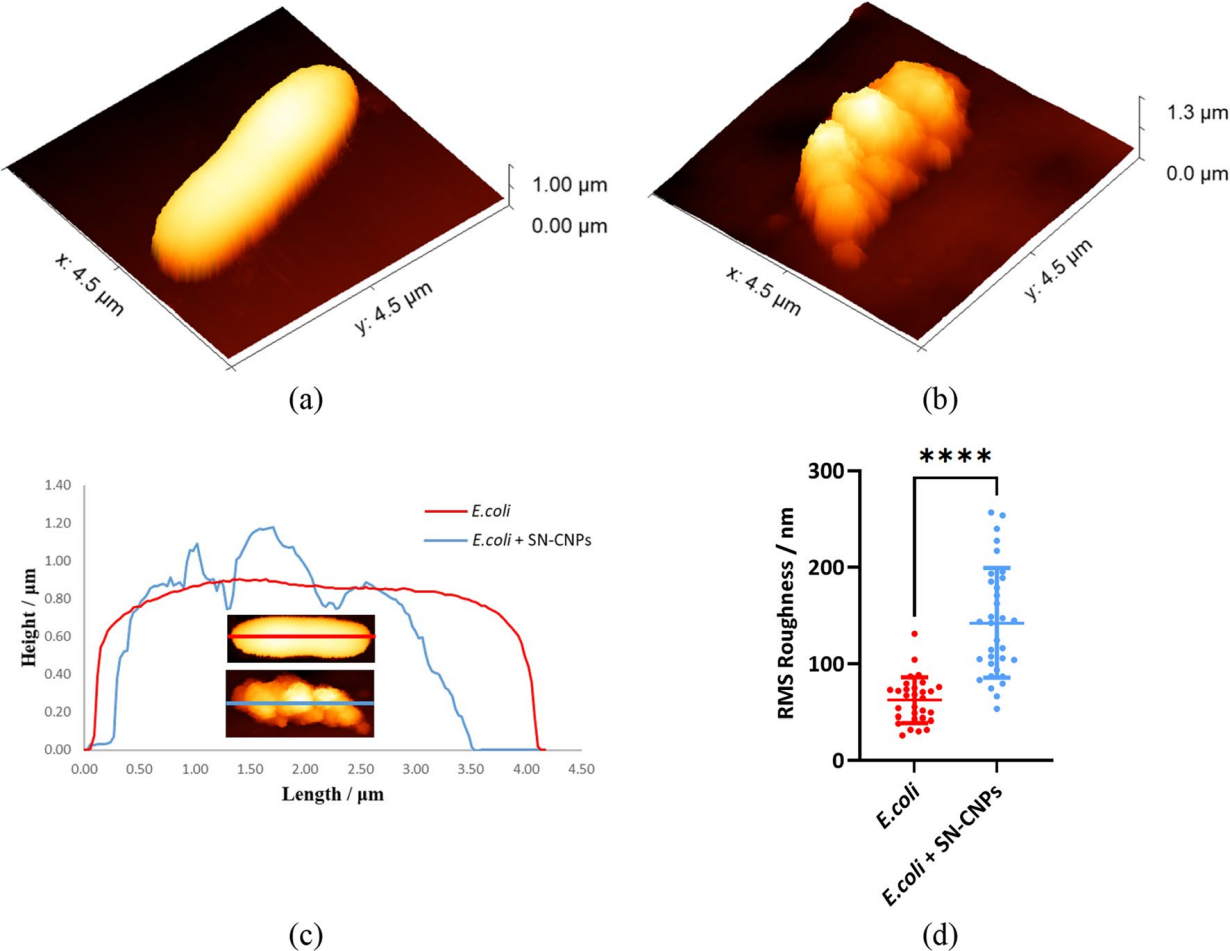
Representative AFM images of live *E. coli* in LB broth before and after treatment of SN-CNPs: **a** 3D image of untreated *E. coli*; **b** 3D image of treated *E. coli* at 200 μg mL^−1^. **c** Height profiles, and **d** Scatter plot showing a significant change in surface roughness after treatment. Error bars indicate the standard deviation obtained from different experiments

To ensure that the observed OM damages of bacteria cells shown in the SEM images were not an artifact of the fixation process or reagent, AFM measurements of live *E. coli* in LB medium for untreated and treated cases were also carried out. Figure 8a, b showed the 3D morphologies of live *E. coli* in liquid culture for control and treated (200 μg mL^−1^) conditions. It can be seen that the surface and shape of SN-CNPS treated *E. coli* was severely altered, having a shorter length and a much larger height variation across the surface, which somewhat recapitulated the blebs previously observed in SEM images. In contrast, the surface height profile of the normal untreated cell was very smooth. To observe and quantify the surface roughness, the height profile of a bisected line drawn along the major axis of the cell was generated (Fig. 8c). The root-mean-square (RMS) roughness of both control and treated cells were calculated and compared in Fig. 8d. The results clearly showed that cells treated with SN-CNPs have significantly higher surface roughness compared to untreated ones, which further confirms our previous observations in fixed cells.

### Quantum mechanical modeling of the interaction between SN-CNPs and phospholipds

**Fig. 9:**
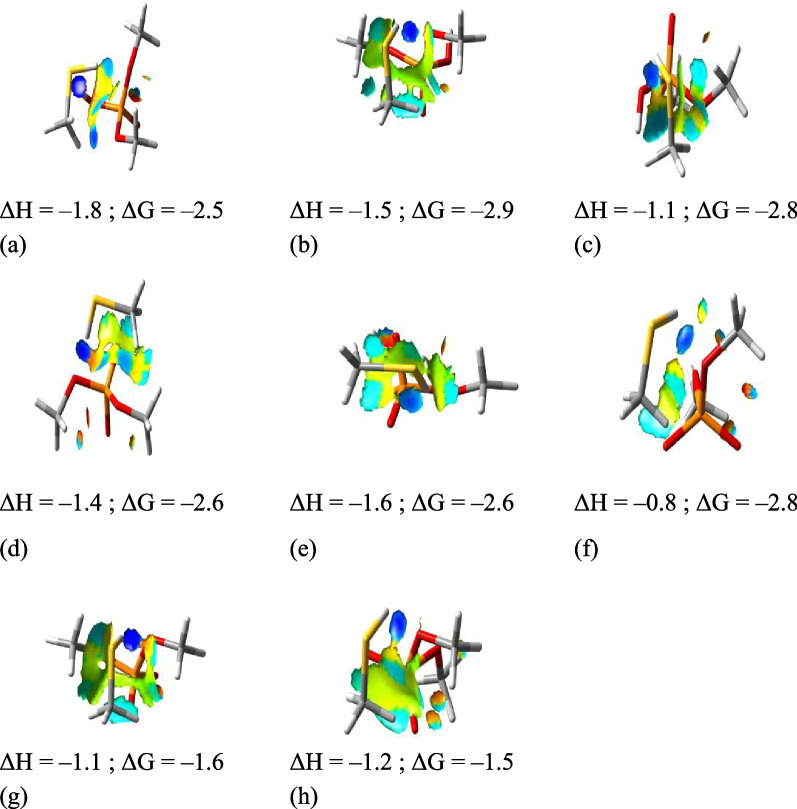
NCIs between MeSH and Me_2_PO_4_H (**a**–**c**) and between MeSH and Me_2_PO_4_^−^ (**d**–**h**) in model dimers. Formation energies at T = 298.5 K and p = 1.00 atm are also depicted (in units of kJ mol^−1^). Level of theory: DFT M06–2X / 6–311++G** // SMD (water)

In order to quantitatively estimate the interaction between SN-CNPs and phospholipid, quantum mechanical calculations were performed using methanethiol (MeSH) to model the key functional group of SN-CNPs and dimethyl phosphate (Me_2_PO_4_H) and its deprotonated form (Me_2_PO_4_^−^) to model the phospholipid. Since the CNPs’ chemical nature and core structure are still undetermined, they were not included in the computational modelling. Despite its simplicity, such an approach was already successfully employed in previous studies [29, 63, 64]. The topological Atoms-in-Molecules (AIM) analysis showed the presence of critical points between the two moieties. The Non-Covalent Interactions (NCIs) between two molecules in different conformations were identified and corresponding formation energies were computed (Fig. 9). NCIs between MeSH and Me_2_PO_4_H indicated some strong attraction (dark blue region) between thiol proton and oxygen in C–O–P in model dimer “b” and between thiol proton and oxygen in P–O–H in model dimer “c”; a weaker attraction (turquoise region) between thiol proton and oxygen in C–O–P as well as between Sulfur and phosphorous was identified in model dimer “a”; a weak attraction between Sulfur and Phosphorous (green region) was also revealed in model dimer “b”.

As for dimers between MeSH and Me_2_PO_4_^–^, the thiol proton exclusively interacted with oxygen in P–O–C. Such attraction was stronger in model dimers “d”, “f”, “g” and “h” than in model dimer “e”. The attraction between Sulfur and Phosphorus was identified in model dimers “d”, “e” and “h”. Me_2_PO_4_^−^ more realistically represented the molecular structure of phospholipid.

The interaction enthalpies and Gibbs’ free energies were negative for all 8 model dimers presented. The thermochemical data confirmed the findings from AIM and NCI analyses; any system with a significant attraction between Sulfur and Phosphorus generally resulted in a more negative enthalpy and free energy than those that do not have this attraction. The AIM and NCI analyses indicated that the dephosphorylation of a phospholipid induced by the thiol group is energetically favorable, especially in neutral or near-neutral pH levels.

**Fig. 10:**
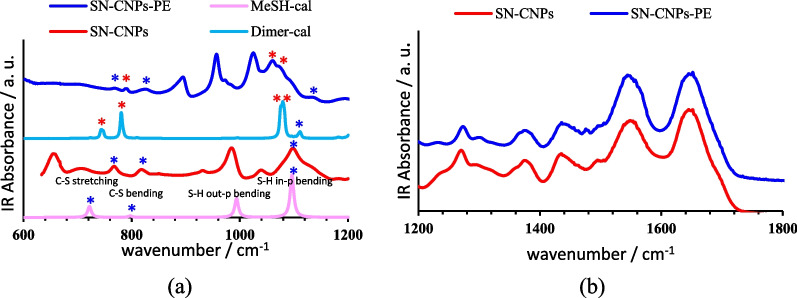
(**a**) FT-IR spectrum of SN-CNPs (red), mixture of SN-CNPs and dimethyl phosphate (blue), calculated IR spectrum of MeSH (pink) and calculated average IR spectrum for dimer between MeSH and Me_2_PO_4_H/Me_2_PO_4_^−^ (cyan) in the wavenumber range from 600 cm^−1^ to 1200 cm^−1^. (**b**) FT-IR spectrum of SN-CNPs (red), mixture of SN-CNPs and dimethyl phosphate (blue) in the range from 1200 cm^−1^ to 1700 cm^−1^. Level of theory: DFT M06–2X / 6–311 +  + G** // SMD (water).

The interaction between thiol hydrate and phosphate ester was further evidenced by the comparison of FT-IR spectra of SN-CNPs and their mixture with dimethyl phosphate through the calculated IR spectra of MeSH and the Boltzmann-like averaged spectrum out of all the potential dimers described earlier between MeSH and Me_2_PO_4_H/Me_2_PO_4_^−^ (Fig. 10a). Characteristic peaks at 764 cm^−1^, 820 cm^−1^, 987 cm^−1^ and 1098 cm^−1^ in the spectrum of SN-CNPs corresponded to the peaks at 722 cm^−1^, 797 cm^−1^, 994 cm^−1^ and 1096 cm^−1^ representing C–S stretching, C–S–H bending, S–H out of plane bending and S–H in-plane bending, respectively (Chandra et al., 2011), shown in the calculated IR spectrum of MeSH. In the average spectrum of the calculated IR for dimers formed between MeSH and Me_2_PO_4_H/Me_2_PO_4_^−^, all the aforementioned characteristic peaks shifted slightly to higher wavenumbers corresponding to 725 cm^−1^, 810 cm^−1^, 996 cm^−1^ and 1111 cm^−1^, respectively, accompanied with a significant decrease in intensity. Meanwhile, some new peaks appeared in this average spectrum at 744 cm^−1^, 780 cm^−1^, 1076 cm^−1^ and 1082 cm^−1^, representing the motion of the S–H group coordinating with the vibrations of Me_2_PO_4_H/Me_2_PO_4_^−^. In the experimental FT-IR spectrum for a mixture of SN-CNPs and dimethyl phosphate, new peaks were observed at 792 cm^−1^, 1063 cm^−1^ and 1070 cm^−1^, corresponding to 744 cm^−1^, 1076 cm^−1^ and 1082 cm^−1^, respectively. On the other hand, no obvious change was shown in the FT-IR spectrum in the wavenumber range from 1200 cm^−1^ to 1700 cm^−1^ (Fig. 10b) upon mixing of SN-CNPs and dimethyl phosphate, indicating that the binding between SN-CNPs and phosphate ester was through hydrate thiol and phosphate groups with little participation of amide groups.

## Conclusions

Sulfur and Nitrogen co-doped carbon nanoparticles SN-CNPs were synthesized, characterized and tested in this study. These particles have a spherical shape with an approximate diameter of 50 nm. SN-CNPs was found to have stronger phosphatase activity than ALP at all three-measured pH values at 4.7, 7.0 and 8.8. To the best of our knowledge, SN-CNPs are the first non-metal based nanozyme possessing phosphatase-like activity. Aqueous solution of SN-CNPs at the concentration as low as 25 μg mL^−1^ showed significant selective antimicrobial effect against *E. coli*. As the concentration of SN-CNPs increased to 200 μg mL^−1^, it also exhibited inhibition against *L. lactis*. SEM images of bacterial cells for different incubation periods after treatment with SN-CNPs suggested the OM was disrupted and the cell wall of both *E. coli* and *L. lactis* was damaged. AFM images of live *E. coli* in LB broth further consolidate our SEM results, indicating that the outer surface of the cell was severely altered when interacting with SN-CNPs, which significantly increased the surface roughness of the affected cells. Thereafter, a bactericidal mechanism based on phosphatase was proposed in the present work. It was hypothesized that dephosphorylation of the phospholipid, the outer layers of OM of *E. coli*, and that of LPS, the major constituents of the cell wall of *L. lactis*, as well as that of phosphate ester, the backbone of *E. coli* cell wall, were mainly responsible for the bactericidal effects of SN-CNPs. Quantum mechanical calculations provided not only strong support for the non-covalent interactions between –SH groups in SN-CNPs and phosphate esters in phospholipids, but also a theoretical foundation for the –SH based dephosphorylation process. Due to their stable fluorescent emission and strong phosphatase activity, this new class of carbon-based nanoparticles can be used for a variety of applications including enhancement of phosphate release, fluorescent labeling of nanomaterials and biological specimens, antimicrobial treatment and targeted cancer therapy.

## Materials and methods

### Synthesis of SN-CNPs

SN-CNPs were synthesized by adding 3 g powdered beet, 2 g element Sulfur powder and 2 g L-cysteine to 24 mL ethylenediamine. The mixture was stirred at 60 °C for 2 h. After adding water to a total volume of 60 mL, the mixture was transferred into an autoclave to undergo a hydrothermal reaction at 200 °C for 5 h. The liquid product was collected through filtration and centrifugation. The yellow-coloured liquid product was left in the hood to be dried at room temperature (upon calibration, the liquid product followed by filtration and centrifugation has the equivalent concentration of 50 mg/mL).

### Characterization

Morphology and size of SN-CNPs were characterized using scanning electron microscope (SEM, Zeiss Crossbeam 540) and JEOL JEM1400 plus transmission electron microscope (TEM). The hybridization status of the atoms distributed on the surface was identified via X-ray photoelectron spectroscopy (XPS, Nexsa, Thermo Scientific) and the surface functional groups were identified using a Thermoscientific Nicolet IS5 FT-IR spectrometer in the range from 500 to 4000 cm^−1^.

### Phosphatase assay and enzymatic tests

Nitro blue tetrazolium/5-Bromo-4-chloro-3-indolyl phosphate (NBT/BCIP) and para-Nitrophenyl Phosphate (pNPP) (purchased from Life Technologies) were used as a substrate. Optical densities at different concentrations of NBT/BCIP and pNPP were measured at 590 nm and 405 nm, respectively. The natural enzyme Calf-intestinal alkaline phosphatase (CIP; from Life Technologies) was used as the positive control, and SN-CNPs/N-CNPs in deionized water as well as the substrates in deionized water were used as negative control.

### Bacterial growth

*Escherichia coli* (Migula) Castellani and Chalmers (ATCC®23,735™) and *Lactococcus lactis* subs derived from ATCC®19,435™ were used in the present work. All bacteria were cultured in lysogeny broth (LB) Lennox medium (Tryptone 10 g, Yeast extract 5 g, Sodium chloride 5 g), and—incubated in a shaking incubator overnight—preliminary bacterial suspensions were obtained.

### Antibacterial activity tests

All bacteria (1 × 10^4^ CFU mL^−1^) were incubated alone and with different concentrations of SN-CNPs (0.01, 0.05, 0.10, and 0.20 mg mL^−1^). After a 2 h-treatment, each bacteria suspension was plated onto LB agar (Tryptone 10 g, Yeast extract 5 g, Sodium chloride 10 g, Agar 12 g) plates. The bacteria colonies formed were counted and recorded after incubation for 16 h at 37 ºC. A single colony of bacteria strain *E. coli* was incubated using 5 mL of Luria–Bertani (LB) medium overnight (16 h) at 37 °C and 250 rpm. The MIC was determined using micro-dilution assay. The microplates were run in a microplate reader at 37 °C. The absorbance at 600 nm was recorded every 30 min for 20 h on a 96 well plate. For each measured well, 50 μL of diluted bacteria was added to 50 μL of SN-CNPs solution diluted in LB broth leading to a final total volume of 100 μL.

### Computational details

Methanethiol (MeSH) and Dimethylphosphate (Me_2_PO_4_H), their de-protonated (anionic) forms, and a series of dimers, were investigated at Density Functional Theory (DFT) level. The (electronic ground state) molecular geometry of the investigated systems was fully optimized, both in vacuo and in the aqueous solution. The results were obtained by employing hybrid viz. B3LYP [65], coupled with the Pople triple-ζ 6−311++ G** basis set. The D3 version of Grimme’s semi-empirical dispersion with Becke–Johnson damping GD3BJ [66] was also included in the case of the B3LYP functional. Solvent effects were taken into account via the implicit polarisable continuum model in its integral equation formalism (IEF-PCM) [67]. The molecular cavity was built using the SMD parameterization [65]. The standard values for dielectric constant and refractive index were always assumed. The thermochemical data, vibrational frequencies and IR peak intensities were computed in harmonic approximation, at T = 298.15 K and p = 1 atm, in high precision mode; no imaginary frequencies were found showing genuine minima for the stationary point achieved upon optimisation. The composition of each vibrational normal mode was analyzed in terms of internal coordinates.

The atomic charges were computed by means of three different approaches, viz. Mulliken’s population analysis, the ChElPG procedure [68] and the atomic polar tensor (APT) derived charges [69–71]. To investigate the presence and nature of possible intermolecular interactions, three different approaches were used: (1) the full Natural Bond Orbital (NBO) analysis [71–75] of the total density (Natural Atomic Charges were also computed); (2) topological analysis based on Bader’s atoms in molecules (AIM) theory [76–78] and (3) the non-covalent interaction (NCI) index combined with the second derivative of the reduced density gradient along the second main axis of variation [79–81]. Hessian of electron density was additionally calculated at along RDG isosurface with |isovalue|= 0.5,

1 $$s=\frac{1}{2{\left(3{\pi }^{2}\right)}^{1/3}}\frac{\left|\rho \right|}{{\rho }^{4/3}}$$

where $\rho$ indicates the total electron density.

For all calculations, the integration grid for the electronic density was set to 250 radial shells and 974 angular points of all the atomic species. Accuracy for the two-electron integrals and their derivatives was set to 10^−14^ a.u. The Self-Consistent Field (SCF) algorithm used a quadratically convergent procedure designed by Bacskay [82], a method which is acknowledged to be slower but more reliable than regular SCF with DIIS extrapolation. The convergence criteria for SCF were set to 10^−12^ for root mean square (RMS) change in density matrix and 10^−10^ for maximum change in density matrix. Convergence criteria for geometry optimisations were set to 2 × 10^−6^ a.u. for maximum force, 1 × 10^−6^ a.u. for RMS force, 6 × 10^−6^ a.u. for maximum displacement and 4 × 10^−6^ a.u. for RMS displacement. For the AIM analysis, the number of paths in each interbasin surface was set equal to 500, the number of points in each interbasin surface path as well as the max number of points of a path equal to 1000, the step size 1 × 10^−4^ bohr, the maximal number of interactions to 512, the criterion for gradient-norm (displacement) convergence to 1 × 10^−7^ a.u. (1 × 10^−8^ a.u.), and the minimal distance between CPs to 1 × 10^−3^ bohr. The search of the CPs was made starting from the nuclear positions, the midpoint of each atom pair, the center of any triangle defined by three atoms and tetrahedron defined by four atoms.

The location of the CPs and subsequent calculation of SF values were performed using a modified version of the PROAIMV program [78, 83]. NCI was computed and the surface plotted using homemade code. All the other calculations were performed using GAUSSIAN G16.A03 package^84^.

### Atomic force microscopy (AFM) measurements

High-resolution AFM images of dried SN-CNPs were measured using the Smart SPM 1000 (AIST-NT, Russia). 6μL of SN-CNPs solution was used to deposit on a new layer of mica for air drying. Images of SN-CNPs were captured using a Super Sharp NSG30_SS AFM probe (Tips Nano) with a typical tip radius of 2 nm, resonance frequency 320 kHz and force constant 40N/m. AC-Mode (tapping mode) was applied with a scanning speed of 0.1 Hz to produce an image with pixel resolution of 1024 × 1024. All AFM measurements of *E. coli* in liquid were performed using Bruker NanoWizard 4XP (Bruker Instruments, Germany) coupled with Zeiss Observer 7 microscope (Carl Zeiss, Germany) for optical imaging. DNP-10 Silicon Nitride probe (Bruker, Germany) with a nominal spring constant K = 0.35 N/m, and a nominal tip radius of 20 nm was used for all measurements. A Contact based method was used to calibrate the sensitivity and the spring constant of the probe. Initially, *E. coli* culture with SN-CNPs for 1 h were poured into a Poly-l-lysine (0.1%) coated Petri dishes and allowed for attachment for 30 min before imaging. For morphological images of cells in liquid (Luria–Bertani medium), QI™ cell in liquid mode with a setpoint of 1.5 nN, resolution of 50 nm/pixel, and a scan speed of 50 μm/sec was used. To obtain statistical averaging, more than 30 cells of both control and treated *E. coli* were imaged. Surface roughness was determined by drawing a line across the center longitudinal axis of the cell to obtain its height profile using the JPK-Data processing application. Finally, the normality and lognormality test, unpaired t-test, and Mann-Whitney test were applied using GraphPad prism 9 for evaluation of the statistical significance.

## Supplementary Information

Additional file 1

## Data Availability

The authors declare that the data supporting the findings of this study are available within the paper and its Supplementary Information files. Should any raw data files be needed in another format they are available from the corresponding author upon reasonable request.
