# Supplementary material for: Novel carbon nanozymes with enhanced phosphatase-like catalytic activity for antimicrobial applications
Source: Discov Nano. 2023 May 23;18(1):76. doi: 10.1186/s11671-023-03856-y (PMC10214917; doi:10.1186/s11671-023-03856-y)
Supplement: Supplementary file 1 — Additional file 1 [file 11671_2023_3856_MOESM1_ESM.docx]

**Supplemental Figures**

| **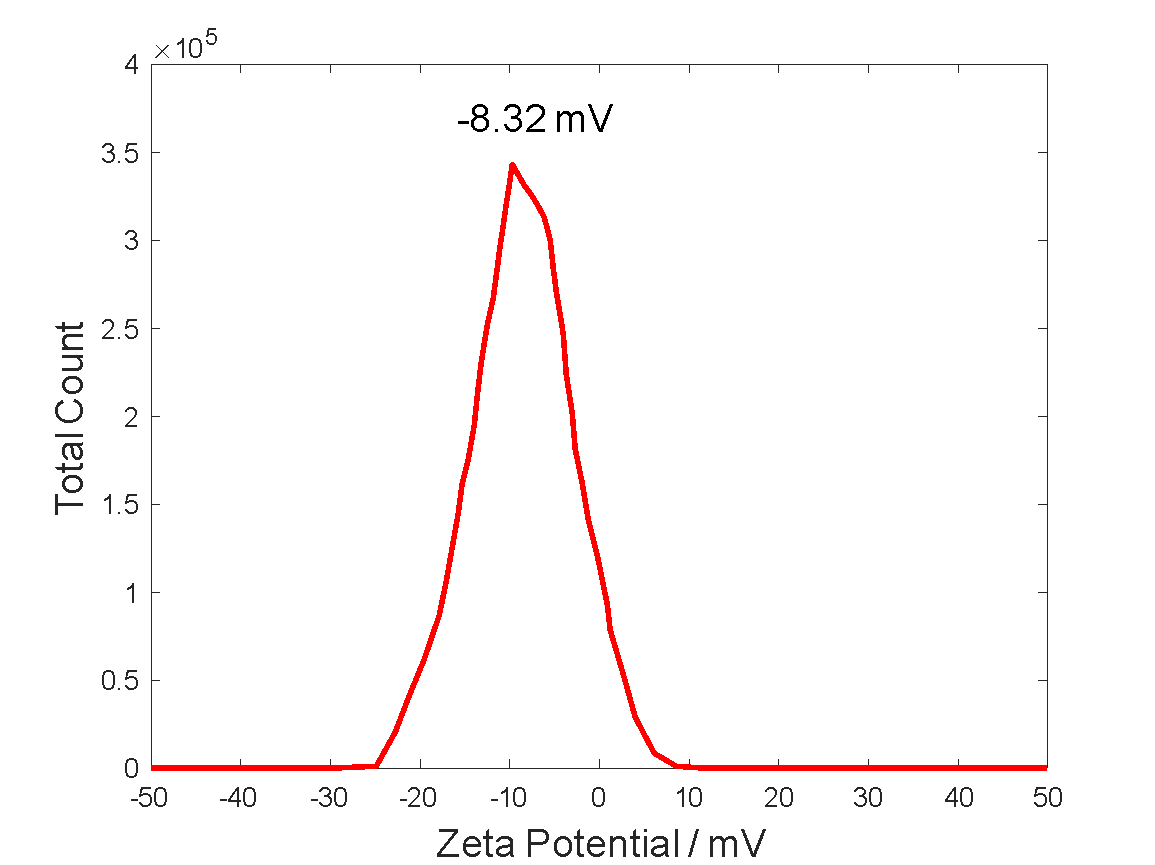**  **Figure S1: SN-CNPs have negative charges.**  The zeta potential of SN-CNPs measured at 50 $\mu$g mL^-1^ indicates that they are negatively charged. 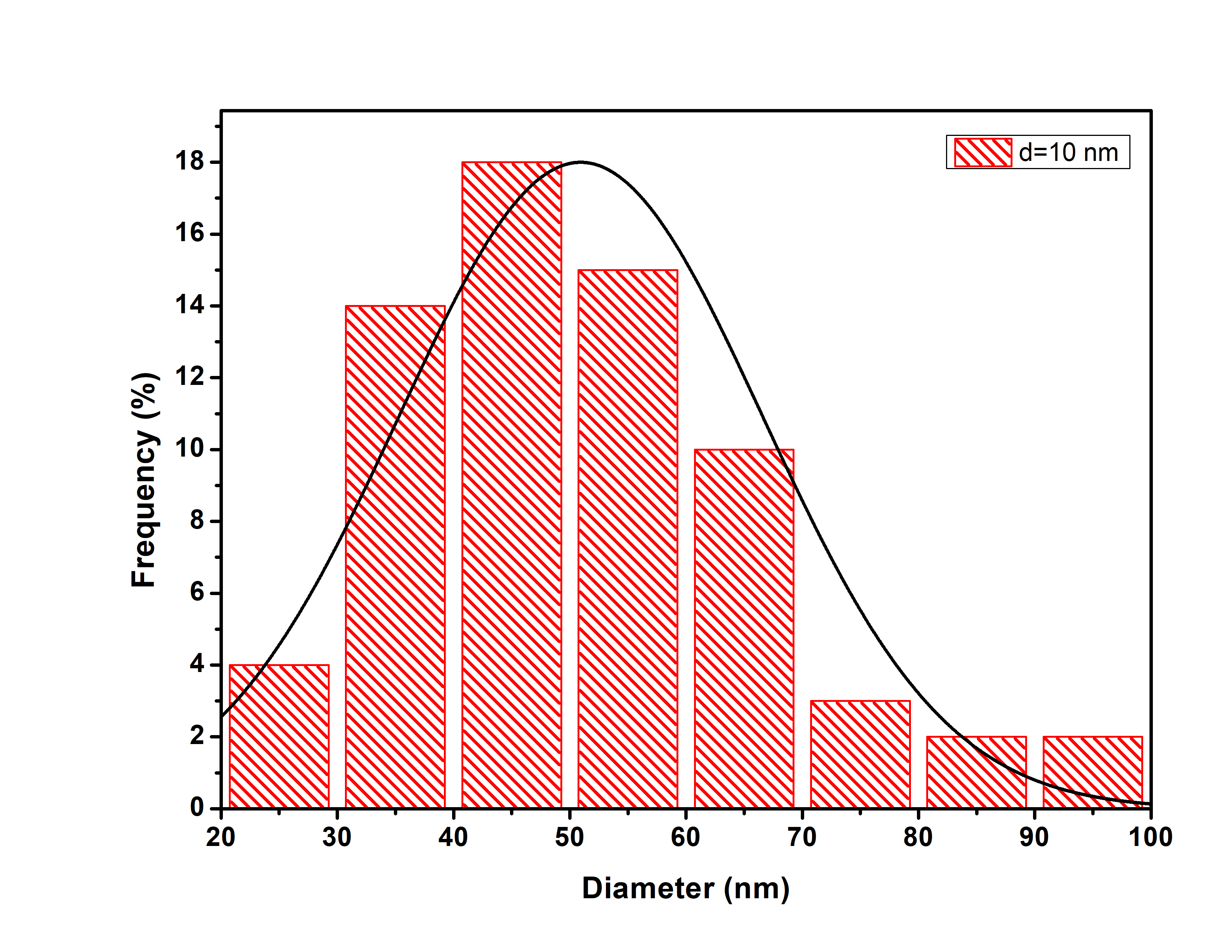 **Figure S2: Size distribution of SN-CNPs.**  Analysis of the TEM images of SN-CNPs measured at 100 $\mu$g mL^-1^ shows that the peak distribution for the diameter of the nanoparticles is around 50 nm. | | |
| --- | --- | --- |
| (a) | (b) |  |


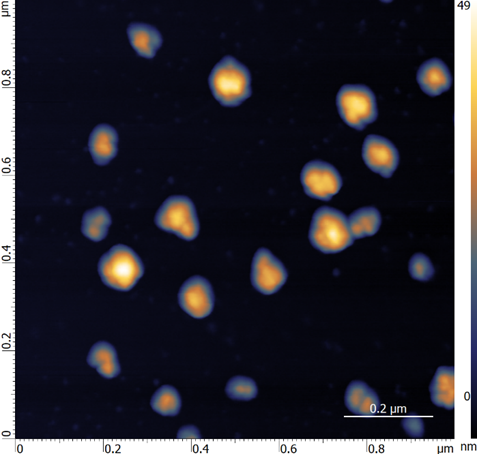

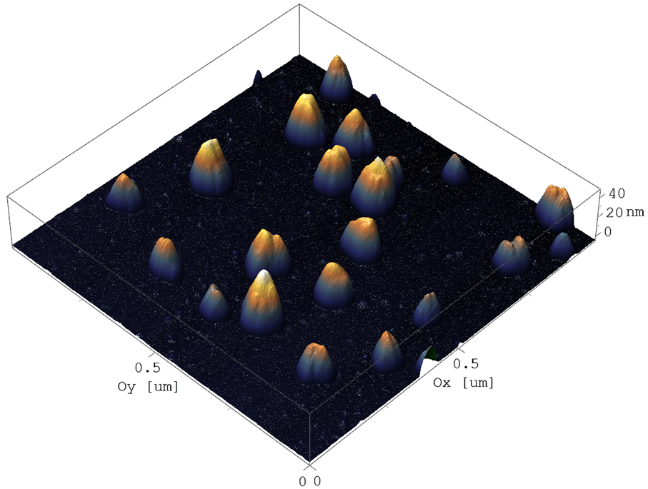


**Figure S3: SN-CNPs form aggregates at high concentration.**

2D (**a**) and 3D (**b**) AFM images of dried SN-CNPs on mica slide show sign of aggregation at 150 $\mu$g mL^-1^.


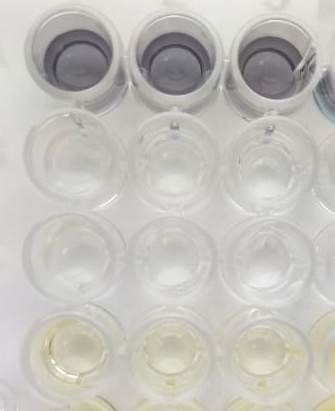

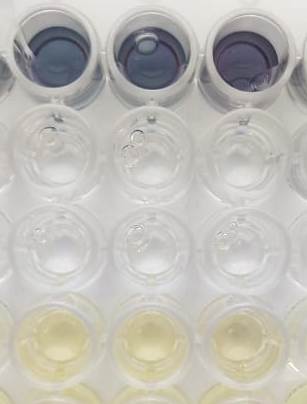

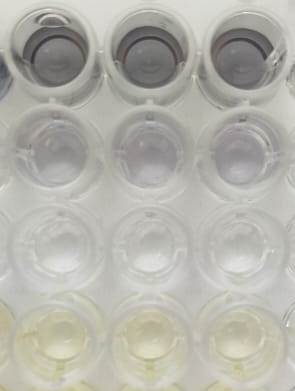


**pH 4.7**

**pH 7.0**

**pH 8.8**

**N-CNP + NBT/BCIP**

**ALP + NBT/BCIP**

**NBT/BCIP control**

**BTSSCT control**

**Figure S4: Phosphatase activity assay for N–CNPs.**

The results show that N-CNPs have low phosphatase activity, especially at pH values that differ from neutral.

| (a) | (b) |
| --- | --- |
| (c)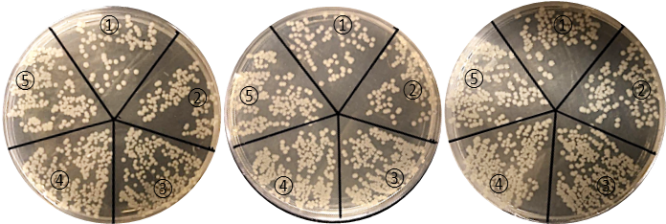 | (d)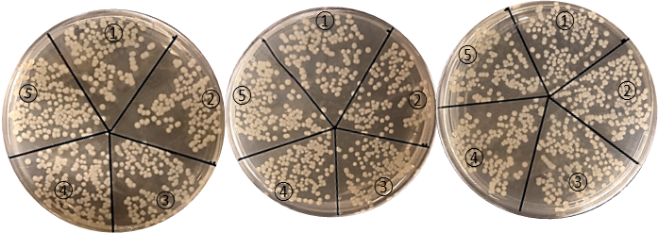 |
| 0 $\mu$g mL^-1^ (1); 10 $\mu$g mL^-1^ (2); 50 $\mu$g mL^-1^ (3); 100 $\mu$g mL^-1^ (4); 200 $\mu$g mL^-1^ (5) | 0 $\mu$g mL^-1^ (1); 10 $\mu$g mL^-1^ (2); 50 $\mu$g mL^-1^ (3); 100 $\mu$g mL^-1^ (4); 200 $\mu$g mL^-1^ (5) |
| (e) |  |

**Figure S5: Antibacterial test for N-CNPs against *E. coli* (left) *and L. lactis* (right).**

Antibacterial activity tests for N-CNPs against *E. coli* (**a, c, e**) and *L. lactis* (**b, d, f**) reveal that N-CNPs have significantly weaker antibacterial properties than SN-CNPs. N-CNPs do not inhibit bacterial growth at concentrations less than 200 $\mu$g mL^-1^, whereas SN-CNPs almost completely inhibit at this concentration.

| (a) | (b) |
| --- | --- |
| 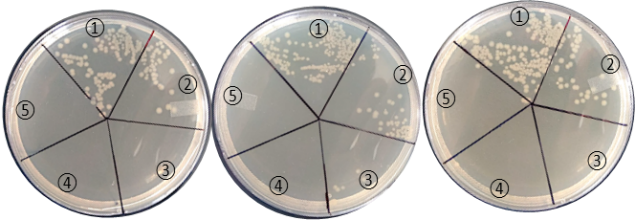  0 $\mu$g mL^-1^ (1); 13 $\mu$g mL^-1^ (2); 25 $\mu$g mL^-1^ (3); 50 $\mu$g mL^-1^ (4); 75 $\mu$g mL^-1^ (5) | 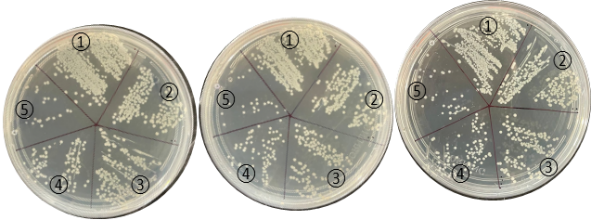  0 $\mu$g mL^-1^ (1); 50 $\mu$g mL^-1^ (2); 100 $\mu$g mL^-1^ (3); 150 $\mu$g mL^-1^ (4); 200 $\mu$g mL^-1^ (5) |

**Figure S6. SN-CNPs have antibacterial properties against both *E. coli* and *L. Lactis*.**

Antibacterial activity tests of SN-CNPs at different concentrations against *E. coli* (**a**) and *L. lactis* (**b**) reveal that *L. Lactis* has much more tolerance or resistance to SN-CNPs than *E. coli*.


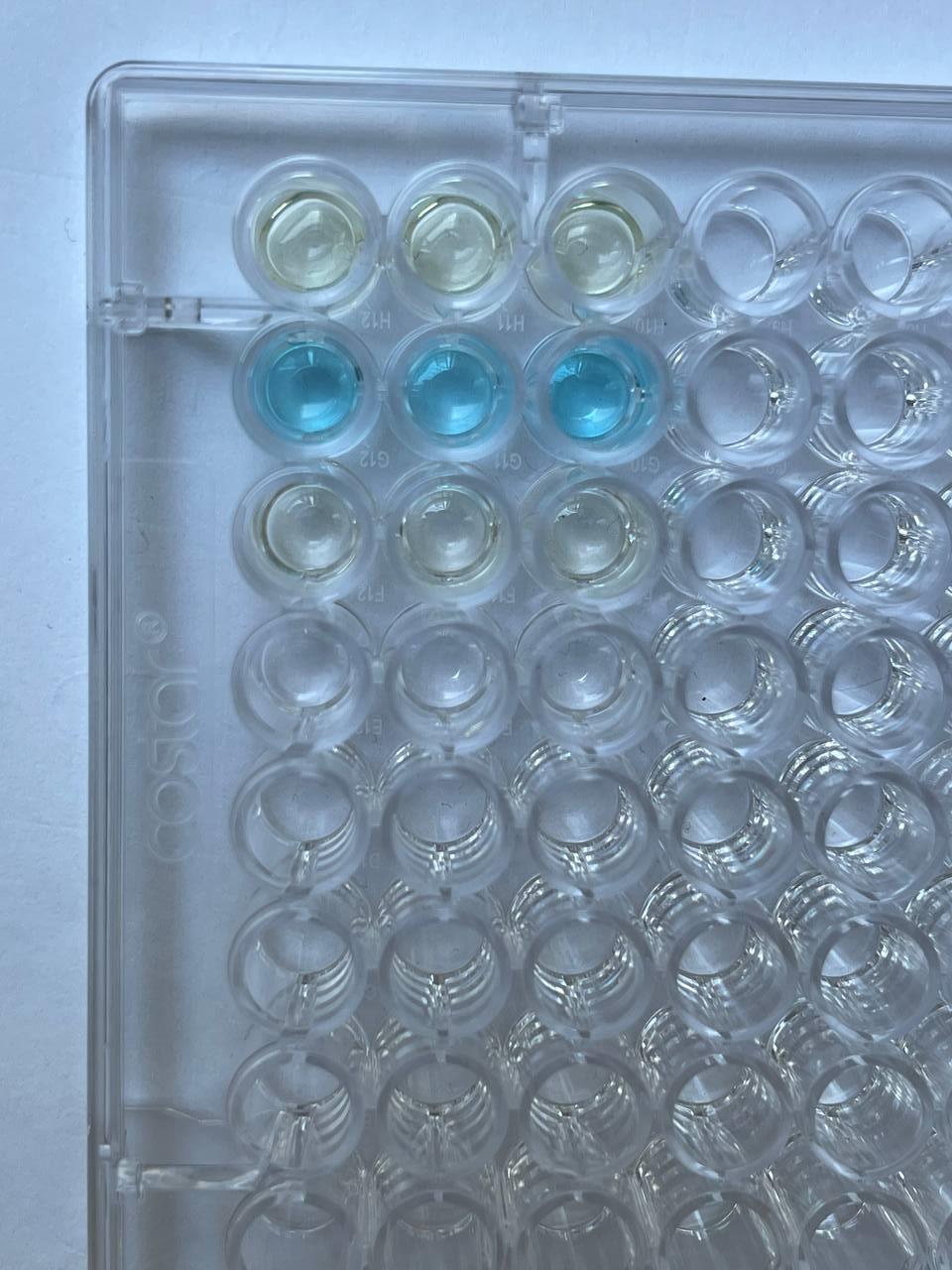


**TMB +SN-CNPs**

**TMB +Horse reddish**

**SN-CNPs**

**TMB**

**Figure S7. Peroxide activity assay for SN-CNPs.**

When using TMB as a substrate, SN-CNPs do not directly induce oxidant activity, whereas horseradish clearly demonstrates the presence of oxidant activity.


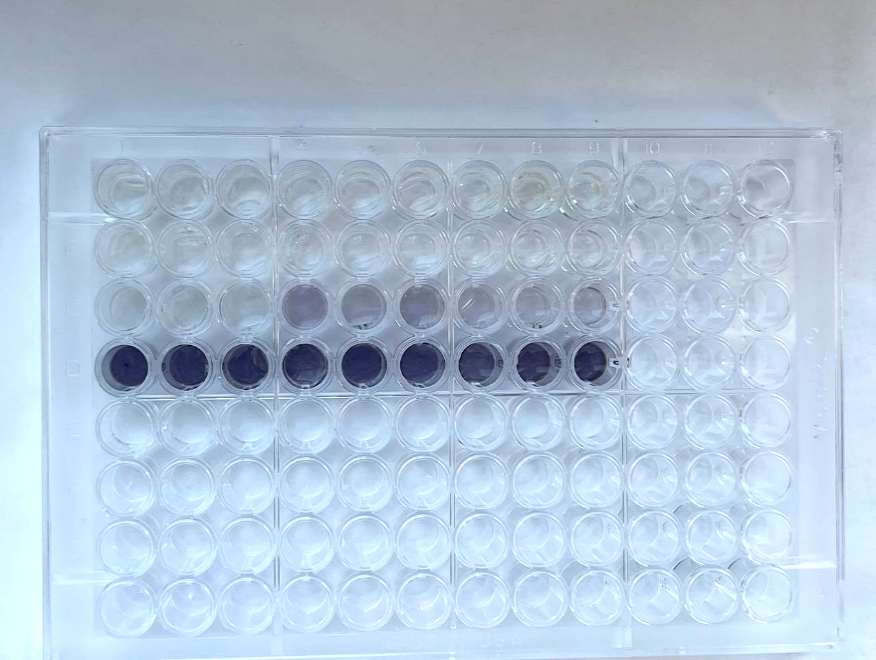


**4.7**

**8.8**

**7.0**

**NBT/BCIP**

**NBT/BCIP+SN-CNPs**

**NBT/BCIP+ALP**

**L- Cysteine**

**NBT/BCIP+L-Cysteine**

**

n.s.

**

**

n.s.

**

**

n.s.

**


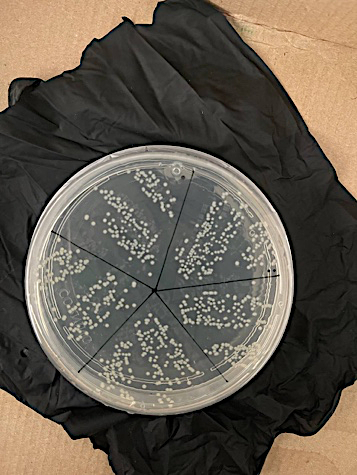

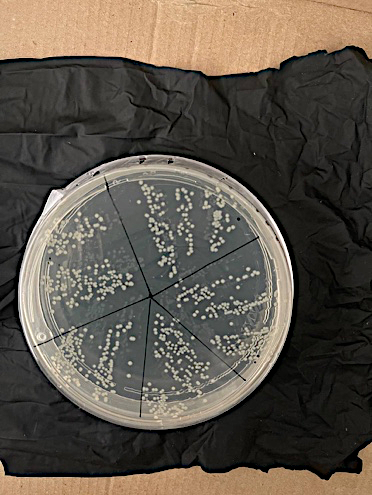

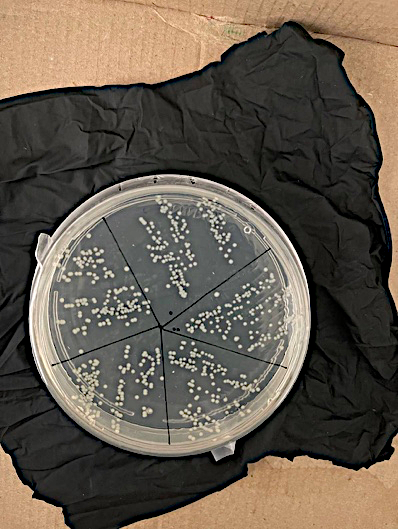


①

②

③

④

⑤

①

②

③

④

⑤

①

②

③

④

⑤

0 $\mu$L (1); 5 $\mu\mu$L (2); 10 $\mu$L (3); 20 $\mu$L (4); 30 $\mu$L (5)

**Figure S8. Phosphatase and antibacterial activity tests for L-cysteine.**

Tests for phosphatase (top row) and antimicrobial activity (bottom row) conclusively show that free cysteine containing the same amount of sulfur as seen in the stock solution of SN-CNPs does not exhibit phosphatase or antibacterial activities. It is important to note that the amount of L-cysteine used here produces exactly the same quantity of sulfur as that found in the SN-CNPs stock solution used in the antimicrobial test against *E. coli*, as shown in Figure 6a.
